# Identity-scaffolded epistemologies: adopting non-normative beliefs online through collective information bias

**DOI:** 10.3389/fpsyg.2026.1843078

**Published:** 2026-07-29

**Authors:** Simon Greipl, Ursula K. Schmid, Diana Rieger

**Affiliations:** Department of Media and Communication, LMU Munich, Munich, Germany

**Keywords:** cavalier humor beliefs, collectivity, conspiracy mentality, gaming, identity-scaffolded bias, online radicalization

## Abstract

Online radicalization thrives not merely on biased individuals but on biased groups. This paper reframes information bias in online communities as a collective epistemology that can sustain non-normative belief dispositions, such as conspiracy mentality and cavalier humor beliefs. We conceptualize identity-scaffolded epistemic style as group-based information bias that is socially sustained by online social identity and homophily. In an online survey of *N* = 827 German-speaking social media users aged 18–30, we asked participants about their use of different online environments as news sources (traditional channels, social media, messenger apps and gaming spaces), thus contextual proxies for social identification and homophily, which in turn were assumed to foster information bias as a core component of recent conceptualizations of echo chambers. Results demonstrate that, across a variety of online platforms and spaces, this identity-scaffolded bias was robustly associated with conspiracy mentality and cavalier humor beliefs, but not anti-migration attitudes, which, in contrast, was correlated with political orientation. This suggests an important distinction between ideology as the content of belief and identity-scaffolded epistemic style as a socially distributed mode of validation. Our findings highlight epistemic biases as a socially embedded mechanism and a potential proximal correlate of online radicalization, foremost in collective online information environments, such as messenger apps and gaming environments.

## 1 Introduction

In contemporary digital ecosystems, belief formation is increasingly shaped by platforms that tie social belonging to how people process information. There is robust evidence that biased information processing is linked to radicalization-relevant beliefs and extremism (Naderer et al., 2024; Simon and Read, 2025; Walzenbach and Hinz, 2025; Wojcieszak, 2010). Prominent frameworks such as motivated reasoning (Kunda, 1990) and ideological rigidity (Zmigrod, 2022) locate these biases within individual cognition. Adopting non-normative beliefs, however, thrives not merely on biased individuals but on biased groups. In extremist groups, it is shared attention and emotion that energize a “political tribe”

(Törnberg and Törnberg, 2024): bias can emerge through collective trust infrastructures and through processes of affective and moral alignment (Atari et al., 2022; Sikder et al., 2020). For example, in low-moderated forums or private messenger groups, COVID-19 conspiracy theories may have gained momentum not through evidence, but through cues of identity performance and collective action (Dow et al., 2021; Frischlich, 2022). While digital echo chambers are sometimes theorized as macro-level architectures (Hartmann et al., 2025), we instead propose an exploratory model of individual psychosocial dispositions associated with highly collective digital spaces. Specifically, we conceptualize a group-based bias as an identity-scaffolded epistemic style, capturing how social belonging and cognition interact in digital communication.

By identity-scaffolded epistemic style, we mean a group-based tendency to maintain coherent worldviews despite contradictory evidence by privileging information congruent with one's social environment. This tendency is socially scaffolded by (a) identification with online communities and (b) homophilic orientation toward like-minded others; these represent socio-affective modes of information processing that rely on identity as an anticipatory and signaling system. We use the term “scaffolded” to denote a concurrent dependency behind the individual's information bias rather than a temporal causal sequence. Rather than deliberate evaluation of evidence, these processes function as context-contingent projection and attribution engines that stabilize knowledge—and thus identity—in self- and group-affirming ways, especially under uncertainty or perceived threat (Hogg, 2004). While these styles may manifest as measurable individual tendencies, we conceptualize them as socio-epistemic habits that can be triggered and sustained by the affordances of the digital environment. This framework helps explain why some socially unacceptable or even radical ideas persist despite weak empirical or moral groundings: they reaffirm identity, generate recognition, strengthen group cohesion and provide validation when external evidence is scarce, contested, or normatively transgressive. We argue that this applies especially to conspiracy mentality and a disposition prevalent in internet culture called cavalier humor beliefs (CHB), a tendency to take even disparaging forms of humor light-heartedly (Hodson et al., 2010). The maintenance and reproduction of both are contingent upon a group or an audience. Because online information engagement often unfolds through association (Dvir-Gvirsman et al., 2024), online news use itself can function as a paradigmatic echo chamber (Carey and Ventham, 2025): identity activation shapes anticipatory priors of information processing and delineates epistemic boundaries. The more identity-laden a space, the greater the likelihood of collective epistemic bias and interpretive segregation where threatening information is reinterpreted or dismissed (Nguyen, 2020).

Reflecting this mechanism where trust trumps the verification of information (Walter et al., 2021), the present study examines how different online spaces used as news sources, including traditional channels, social media, messenger and gaming environments, relate to conspiracy mentality, anti-migration attitudes, and CHB through identity-scaffolded information biases. In contrasting these outcomes, we explore a distinction between ideology (what is believed) and epistemic style (how beliefs are socially validated) as a differential mechanism relevant to sustaining or adopting beliefs in online information environments.

## 2 Online political communication as an identity-reinforcing social practice

Information seeking has shifted toward online spaces (Newman et al., 2024), where an “outrage industry” (Berry et al., 2016) increasingly undermine traditional gatekeeping (Van Aelst et al., 2017). In high-choice environments, users rely on individual and group-based heuristics to navigate political content, shaped by social media logics that can foster problematic news habits such as algorithmic dependence or “news-finds-me” orientations (Bakshy et al., 2015; Gil de Zúñiga and Diehl, 2019; see also Kümpel, 2022). These can in turn amplify technologically driven biases, such as algorithmic filtering or recommendation systems fostering distorted impressions of extremist material and social consensus (e.g., on Youtube; Whittaker et al., 2021), or by appeal to cognitive biases, for instance by making radical messages appear more convincing or familiar (Bouko et al., 2022).

However, from an affordance perspective, the perceived network through which news is shared and received, makes “News use on social media […] a social practice” (Dvir-Gvirsman et al., 2024). Keipi et al. (2016) describe this mechanism in their Identity-Bubble Reinforcement Framework: online news use constitutes a social practice of identity reinforcement, where selective exposure and sharing express and sustain belonging. Over time, algorithmic curation can again amplify these preferences, producing identity bubbles. These are self-reinforcing epistemic spheres in which attitudes and perceptions grow increasingly homogeneous, fostering polarization and epistemic segregation.

According to Kaakinen et al. (2020), three factors drive these bubbles: social identity, homophily, and information bias. Identity anchors users in online groups that shape self-concept and belonging (Tajfel and Turner, 1979); homophily fosters ties with similar others, reducing exposure to diverse views (McPherson et al., 2001; Zollo et al., 2017); and information bias sustains selective exposure and trust in in-group sources (Flanagin et al., 2014; Messing and Westwood, 2014). Together, these dynamics create socio-affective environments that reward identity-congruent reasoning (Kaakinen et al., 2020). This aligns with the Social Identity Perspective in communication science, which holds that salient group identities guide categorization, reasoning, and media selection in identity-consistent ways (Hornsey, 2008; Slater, 2007).

Empirical evidence supports this link. (Pearson and Knobloch-Westerwick 2019) showed that partisan participants displayed stronger confirmation bias in online media environments than in print, as digital formats enhance self-directed, identity-based selectivity. Within such online environments, political discourse functions as a form of identity enactment rather than rational exchange (Lane et al., 2026), characterized as a “game of insults and provocations” (Anderson, 2021, p. 14; see also Kahan et al., 2007) that prioritizes group signaling over rational deliberation.

## 3 Radical collectivity across platforms: validation first, information later

How partisanship and identity shape online information processing and how “…peers are likely to invest blind faith in the content shared on social media groups without subjecting it to verification” (Shareef et al., 2020, p. 1) may resemble what Simon and Read (2025) term coherence-based reasoning on a group level—a preference for group coherence over accuracy.

We argue that digital environments enable this coherence-based reasoning within groups through the affordance of collectivity. Collectivity refers to the possibility to form and manage groups online and to experience identity collectively, which can amplify radicalization (Schulze et al., 2024). From a strategic perspective, for instance, Gong (2026) found that the same far-right actors used a variety of platforms for dedicated purposes. While Twitter/X was used to publicly interact with and confront mainstream journalists and politicians (framed as the elites), Telegram's opportunity structure created the communicative hub for their ideological core activity (Buehling and Heft, 2023; Schulze et al., 2022; Zehring and Domahidi, 2023).

Importantly, online environment affordances vary along four structural dimensions: collectivity, moderation intensity, algorithmic curation, and identity salience. Traditional online news outlets represent highly regulated institutional environments where content adheres to editorial standards, moderation is transparent, and algorithmic curation is low. Consequently, collectivity and identity salience remain low; news consumption is largely individual, anonymous, and detached from community architectures. Social media platforms occupy a structurally distinct position characterized by inconsistent, algorithmically mediated moderation that prioritizes engagement over accuracy. Behavioral curation creates personalized information streams that reinforce pre-existing preferences. While showing internal heterogeneity regarding personalization and network association (Sude and Dvir-Gvirsman, 2023), these spaces exhibit moderate collectivity and identity salience. Platforms like TikTok or X reward performative belonging via emotional amplification, utilizing features such as likes, comments and emojis that help users in building “agentic communities, consensualize around normative understandings, and align mutually shared emotions” (Lüders et al., 2022, p. 7).

Conversely, messenger platforms (e.g., WhatsApp, Telegram) utilize relational architectures that foster epistemic trust through familiarity. Here, moderation is minimal, algorithmic filtering is absent, and information flows directly through social ties. Collectivity is high (Schulze et al., 2024): communication is frequently embedded in small, often trust-based interpersonal networks that provide social scaffolding for group-based identification and homophilic exchange. Identity salience is activated directly through this social context, where news use serves socialization as much as informational motivations, acting as a relational catalyst for conversation (Lou et al., 2021). This highlights the communicative and relational value of news consumption in these environments, which may further reinforce the socio-epistemic orientations. Gaming environments share several features with messenger platforms, including low moderation, minimal algorithmic curation, and high collectivity through community-oriented interaction. This affect-laden, informal culture is particularly susceptible to identity activation and the entrenchment of in-group epistemic norms. Gaming spaces fuse play with group belonging (Gee, 2007; Kowert et al., 2022), creating boundary-enforcement dynamics that can be exploited for identity maintenance and validation (Newhouse and Kowert, 2025).

Taken together, messenger and gaming platforms combine emotional immediacy and anonymity with minimal moderation, forming radicalization-prone infrastructures (Kakavand, 2024; Schulze et al., 2024; Urman and Katz, 2022). These spaces illustrate how hostility can proliferate through audience reinforcement (Jiang et al., 2023), frequently driving online hostility less by deviance than by social gratification, such as approval, visibility, and belonging (Walther, 2024). These dynamics generate synchronized group affect and shared validation (Törnberg and Törnberg, 2022), adapting to the specific affordances of each platform (Phadke and Mitra, 2024).

We treat these broader environments as sharing recurring structural patterns that are theoretically relevant to the mechanisms under investigation, particularly with respect to collectivity and identity salience. Individual platforms within these categories differ significantly from one another, for instance in terms of the exact degree of moderation, algorithmic curation, or collectivity they afford. Likewise, usage patterns are highly individualized and may vary across time and specific media contexts. Nonetheless, it can be theoretically assumed that, particularly in relation to news use, these broader environments share the above-mentioned recurring structural patterns.

In environments where collectivity facilitates socio-affective dynamics, beliefs grounded in social rather than factual coherence thrive, even in the face of moral or ethical flaw. Conspiracy mentality and CHB may exemplify the potential of a validation-dependent epistemology. While often explained as the product of individual traits such as need for closure (Webber et al., 2018), epistemic vice (Meyer et al., 2021), or an overreliance on intuition and a lack of reflection (Binnendyk and Pennycook, 2022), conspiracy mentality might also be a socially embedded epistemic style for managing uncertainty. They fulfill epistemic (coherence), existential (control), and social (belonging, distinctiveness) motives simultaneously (Douglas et al., 2017), transforming uncertainty into collective purpose (Hornsey et al., 2025) by projecting intentionality and malevolent agency onto the world (Imhoff and Bruder, 2014).

A similar mechanism applies to CHB, the disposition to treat even disparaging humor as harmless and to dismiss its negative outcomes on others (Hodson et al., 2010). Humor operates as a social calibration mechanism, signaling shared norms through laughter (Billig, 2005). The satisfaction lies in mutual recognition: humor operates as identity performance, reinforcing cohesion and moral immunity (Attardo, 2023; Shifman, 2013). Without affirmation, the joke may collapse into offense. This dynamic is particularly pronounced in digital media environments, where humor frequently circulates in the form of memes, whose defining features lie in their collectivity, adaptability and rapid dissemination through iterative sharing (Shifman, 2013). Hodson et al. (2010) demonstrate that individuals with stronger CHB view humor as serving both affiliative (enhance relationships and in-group cohesion) and aggressive functions (express hostility toward an outgroup). Moreover, CHB are more pronounced among conservative individuals and mediate between their political identity and the endorsement of disparagement humor (Buie et al., 2022).

From this perspective, various attitudinal tendencies can potentially become identity-scaffolded. For instance, when politicized through populist discourse, populist actors recast migration as a zero-sum out-group threat (“they take our jobs, they change our culture”), thereby converting a perceptual cue into an identity-protective cognition (Kahan, 2017). Within messenger groups, gaming guilds, or algorithm-curated feeds, social identity activates the out-group as a salient target, while homophily ensures that users are repeatedly exposed to migration-negative content. This configuration yields an information-bias filter that preferentially selects and amplifies threat-laden frames (Kaakinen et al., 2020; Whittaker et al., 2021). Consequently, homophilic networks on Twitter and Facebook dominate online interactions (Cinelli et al., 2021) and anti-refugee sentiment on Facebook predicts crimes against refugees (Müller and Schwarz, 2020).

However, we argue that anti-migration attitudes do not inherently require the same level of socio-epistemic scaffolding as conspiracy theories. While the latter are often empirically unfalsifiable and thus rely on group validation to remain viable, anti-migration sentiments may be anchored in stable personality orientations. These attitudes originate from distinct motivational systems rooted in externally verifiable instances in the “real world” (Duckitt, 2001; Hjorth, 2020; Meuleman, 2018). Prejudice against migrants can thus be anchored in salient social categories and everyday experiences of perceived cultural threat or economic competition. This provides a form of self-sustaining validation less dependent on communal or epistemic scaffolding—unlike conspiracy theories, which are unfalsifiable and thus require the prosthetic support of the group to remain psychologically viable. Thus, as a convergent ideological pillar of right-wing political orientations in Europe (Halikiopoulou and Vlandas, 2020; Rydgren, 2008) related to persistent constructs such as Social Dominance Orientation or Right-Wing Authoritarianism (Cohrs and Stelzl, 2010; Kleppesto et al., 2024), such attitudes may not require constant social reaffirmation. Our empirical model thus anticipates that epistemic style (identity-scaffolded group-bias) is the primary driver for conspiracies, while stable ideology is the primary driver for anti-migration attitudes. This suggests an important distinction between dispositions mediated through socio-epistemic styles and those anchored in more stable personality or value orientations.

## 4 News use and identity-scaffolded group bias: gradients of online echo chambers?

Echo chambers, frequently discussed in political communication, reflect socially sustained epistemic environments rather than individual irrationality (Nguyen, 2020; Turner, 2023). In Nguyen's (2020) view, echo chambers are architectures of trust and exclusion that reward internal coherence while discrediting external evidence. Yet empirical detection remains notoriously inconsistent due to varying conceptualizations and data access (Hartmann et al., 2025).

Neither homophily (Hartmann et al., 2025) nor identity alone seem to guarantee the formation of echo-chambers or the adoption of radical belief dispositions. In their analysis of political Twitter networks in Poland and Hungary, (Matuszewski and Szabó 2019) found that while partisan identity predicted some clustering, communication patterns were also shaped by media systems, elite signaling, and platform design. Gradients of echo-chambers were found along partisan, ideological, or even platform-specific lines (Di Martino et al., 2025). This variance may exist because echo chambers represent *social epistemic structures* that reinterpret rather than simply ignore threatening information (Nguyen, 2020). Consequently, they rarely appear as hermetic clusters or as automatic outcomes of shared identity. While identity and homophily provide the socio-affective foundations, the epistemic boundaries around extremist ideologies often require reinforcement through communicative (and technological) feedback (Kossowska et al., 2023; Törnberg et al., 2021). Extending the work of Kaakinen et al. (2020), we conceptualize information bias as *socially scaffolded* by identity and homophily. Specifically, while the Identity-Bubble Reinforcement model conceptualizes social identity, homophily, and information bias as a correlated cluster that characterizes an online bubble, our framework arranges these factors into a directional socio-epistemic mechanism. We conceptualize information bias not as a parallel feature, but as a dependent epistemic style that is actively scaffolded by identity and homophily. Together with platform affordances that enable this social scaffold through collectivity, these factors form the psychological and technological opportunity structure for cultivating non-normative group attitudes and adapting radical belief dispositions.

A central observation is that bias is mostly reinforced, sustained, or activated by social dynamics rather than emerging in isolation. Individuals tend to overestimate attitudinal consensus (Nickerson, 1999), apply asymmetric to in- vs. out-group behavior (Hewstone et al., 2002), and avoid belief formation that might jeopardize their belonging or support within valued groups (Cohen, 2003). As (Kahan 2017, p. 2) notes, individuals develop “habits of mind” that align belief formation with identity-defining affinities, cultivating epistemic styles that preserve social belonging and coherence rather than factual accuracy. While these habits can be measured as stable psychological dispositions, our model treats them as state-like epistemic responses to collectivity. This suggests that the style is not merely an internal trait, but a relational dependency that requires constant social scaffolding to remain active. Evolutionary accounts suggest that such biases once served adaptive purposes for learning within social groups (Park, 2022), as imitation was historically safer than experimentation. Thus, human cognition evolved to privilege identity-relevant over ambiguous information. From a predictive-processing perspective, identities function as priors that constrain the interpretation of incoming information (Clark, 2013; Friston, 2010; Hohwy, 2020); when such priors are affectively stabilized within groups, they yield identity-scaffolded information bias—epistemic styles privileging social coherence, affiliation, and validation over accuracy. We distinguish identity-scaffolded epistemic style from established frameworks of motivated reasoning and identity-protective cognition (IPC) by shifting the focus from individual cognitive desire to socially distributed validation architectures. While motivated reasoning posits that individuals process information to reach a preferred conclusion (Kunda, 1990), identity-scaffolding suggests that this preference is not merely an internal drive but is outsourced to the group. Furthermore, identity-scaffolding diverges from the generalized framework of IPC (Kahan, 2017) along two dimensions: the locus of cognitive effort and environmental dependency. While IPC describes an active, resource-intensive defense where individuals deploy reasoning proficiency to counter-argue threatening facts, identity-scaffolding describes a heuristic process of epistemic outsourcing. The individual defers to the homophilic network's validation rather than (necessarily) performing active cognitive labor. Additionally, whereas IPC suggests this protective habit as an internalized trait applied to information across contexts, our framework posits an environmental dependency, such as the bias is structurally co-created and sustained by the specific affordances of the digital space.

Online news use exemplifies these dynamics as paradigmatic echo-chambers (Carey and Ventham, 2025). Social platforms create socio-epistemic structures—networks and algorithmic feeds—organized around shared beliefs, amplifying them through selective exposure and trust manipulation. While echo-chamber theory emphasizes structural containment, our identity-scaffolded model specifies the anticipatory mechanism: how identity salience and homophilic ties preconfigure what appears credible or meaningful before evidence is processed. Thus, unlike cognitive models, our framework accounts for the social validation dependence of belief, in which accuracy is sacrificed to maintain the external social scaffold that stabilizes the individual's world.

Crucially, identity-scaffolded bias differs from ideology. Ideology structures content—what individuals believe (Jost, 2009)—whereas epistemic style structures *form*—how information is processed and validated. While political orientation colors evaluation, it does not necessarily account for the anticipatory filtering that guides reasoning (Kahan, 2013). Empirically, ideological extremity alone is a poor predictor of deviant offline behavior, such as participation in illegal protests (Zhang et al., 2022). Modern partisanship instead reflects social sorting, the fusion of racial, religious, and ideological identities into a single partisan self (Mason and Wronski, 2018). Online radicalization thus progresses less through persuasion than through collective epistemic synchronization—the consolidation of identity-scaffolded information bias (Bouttier et al., 2024).

In sum, identity-scaffolded bias is not a reasoning flaw but a socio-affective adaptation: a social epistemic style that stabilizes meaning, belonging, and coherence under uncertainty. Ideology organizes what is believed (Burs et al., 2023), identity scaffolding governs the validation (Van Bavel et al., 2024). Digital affordances, by amplifying identity and homophily, facilitate this bias and thereby potentially increasing the individual's susceptibility to adopting and maintaining radical belief dispositions.

## 5 The current study

To approximate this mechanism empirically, we estimated a sequential mediation model linking online news use among German young adults (*N* = 827), identity-scaffolded bias, and attitudinal outcomes. Online news use serves as a contextual antecedent interpreted here as an individual level indicator of exposure to socially sustained epistemic structures that vary across digital environments. We assume that digital spaces low in moderation and algorithmic curation, but high in collectivity (e.g., interpersonal proximity and affective immediacy) are particularly conducive to identity activation and homophilic orientation. While messenger apps rely on social scaffolding (interpersonal trust), social media platforms (TikTok/Instagram) rely additionally on algorithmic scaffolding (engagement-based filtering). This may lead news consumers on social media toward information bias more directly, potentially bypassing the prerequisite of explicit social identification. Rather than observing affordances directly, we treat four broad news environments as structural proxies that differ systematically in collectivity, moderation, and identity salience: gaming environments and messenger apps represent environments low in moderation and algorithmic curation and high in collectivity as opposed to social media and traditional news media. We interpret identity and homophily as psychological correlates of, and contributors to, these epistemic structures rather than as direct measurements of network topology. Finally, we propose that identity^1^ and homophily jointly fuel group-based information bias—a socio-affective mode of reasoning that we assume privileges coherence and validation over accuracy. This group-based bias is modeled as a proximal mediator of three attitudinal dispositions: conspiracy mentality, cavalier humor beliefs, and, for contrast, anti-migration attitudes^2^. Robust links to radicalization exist especially with respect to conspiracy mentality (Adam-Troian et al., 2021; Imhoff and Bruder, 2014) and intergroup hostility (Allington et al., 2023). Conspiracy mentality is less tied to a specific ideology (Bartusevičius et al., 2020), while anti-migration attitudes are consistently associated with radical right orientations (Halikiopoulou and Vlandas, 2020). Related to Social Dominance Orientation (Hodson et al., 2010) CHB may be more overtly relevant to radicalization dynamics by supporting the normalization of non-normative ideas through, for instance, reducing the perceived harmfulness of online hostility (Schmid and Greipl, 2025). Importantly, we categorize them as radicalization relevant non-normative belief dispositions, as none of the three constitutes radicalization proper.

The model tests whether distinct information environments afford differing associations with socially scaffolded epistemic styles, and whether these styles explain the variance in belief dispositions. Building on Kaakinen et al. (2020) insights into shared identity and selective validation, we assess how identity-driven anticipatory filtering translates into cognitive and attitudinal outcomes across (online news) contexts differing in collectivity. We expect these mechanisms to operate most strongly for beliefs contingent on external social reinforcement—conspiracy mentality and cavalier humor beliefs (CHB). Finally, we contrast identity-scaffolded bias with political orientation, which we assume structures the content of what individuals believe (Jost, 2009) but not necessarily the form of cognition that determines what counts as credible or salient.

In summary, we propose three hypotheses:

H1: Social identity and homophily will be associated with group-based information bias, thereby scaffolding identity-based information bias.

H2a: Group-based information bias (as identity-scaffolded via social identity and homophily) will exhibit larger associations with conspiracy mentality and CHB than political orientation.

H2b: Political orientation will exhibit larger associations with anti-migration attitudes than identity-scaffolded information bias.

H3: Less moderated, identity-salient online environments (messenger and gaming) (a) show stronger associations with social identity and homophily and show stronger (b) indirect associations over group-information bias to validation-dependent belief dispositions such as conspiracy mentality and cavalier humor beliefs than traditional or social media.

## 6 Methods

### 6.1 Participants and data collection

We focused on young adults aged 18–30, (Schmid and Greipl, 2025) a group likely to use diverse online news spaces deliberately and to participate in the gaming online ecosystem (Greipl et al., 2025). Participants were recruited in Germany and compensated by the survey institute Bilendi/respondi, following a quota-based approach with quotas for age, gender, residence and education. The study is therefore situated within the German socio-political context, characterized by comparatively strict regulation of online hate and platform governance under EU regulations, in contrast to contexts such as the United States, which are more strongly shaped by free speech protections. After removing 53 respondents for quality reasons, the final sample consisted of *N* = 827 social media users (age *M* = 24.38, *SD* = 3.55; 49% female, 55% with higher education, 18% with a migration background). All participants provided informed consent, and the study was approved by the Research Ethics Committee of the Faculty of Social Sciences at the LMU Munich (GZ 24–03).

### 6.2 Measures

#### 6.2.1 Political orientation

Political orientation was measured on a 10-point left–right self-placement scale (1 = far left, 10 = far right), with a mean of 5.77 (*SD* = 2.21).

#### 6.2.2 Media and news use

Participants reported their frequency of use across major online communication platforms on a 5-point scale (1 = never, 5 = several times a day). Media use was highest for WhatsApp (*M* = 4.61, *SD* = 0.75), followed by Instagram (*M* = 4.05, *SD* = 1.23). Moderate use was observed for TikTok (*M* = 3.03, *SD* = 1.67) and Facebook (*M* = 2.71, *SD* = 1.54). Telegram (*M* = 2.07, *SD* = 1.36), Discord (*M* = 2.09, *SD* = 1.34), and Steam (*M* = 2.04, *SD* = 1.32) were less frequently used, including other gaming-related platforms and forums (*M* = 2.04, *SD* = 1.32).

Participants were then asked to report how often they used four different online environments as news sources (“Which channels and platforms do you use to inform yourself about the news?”; 1 = never, 5 = very often). The most common sources were social media platforms (e.g., TikTok, Instagram, Facebook; *M* = 3.57, *SD* = 1.24), followed by traditional news outlets (e.g., online- and offline newspaper, radio, TV; *M* = 3.17, *SD* = 1.21). Messenger apps were used moderately as news sources (e.g., WhatsApp, Telegram; *M* = 3.00, *SD* = 1.55), while gaming platforms and forums were least often used for news consumption (e.g., Discord, Steam; *M* = 2.10, *SD* = 1.38).

#### 6.2.3 Identity and bias constructs

We used the German adaptation of the Identity Bubble Reinforcement Scale (IBRS; Kaakinen et al., 2020) to measure the three constructs as socio-affective mediators using 5-point agreement scales (1 = strongly disagree, 5 = strongly agree). *Social Identity* (3 items; *M* = 2.86, *SD* = 1.28; α = 0.87) captured a sense of belonging and pride in one's online or social communities. *Homophily* (3 items; *M* = 3.51, *SD* = 1.04; α = 0.79) reflected preference for interaction with like-minded others and value alignment. *Information Bias* (3 items; *M* = 3.29, *SD* = 1.05; α = 0.59) assessed perceived credibility and relevance of information that aligns with one's social milieu. While the IBRS measures individual-level tendencies, we interpret these as proxies for the internalized epistemic rules that govern group-based information processing. We acknowledge that while these tendencies are measured at the individual level, they represent the psychological residue of collective epistemic synchronization. Please see Kaakinen et al. (2020) for the original scale. The German adaptation used in the current study can be found online.

#### 6.2.4 Non-normative belief dispositions

Three outcome variables captured belief systems associated with identity-based epistemic styles. *Conspiracy Mentality* (4 items; α = 0.81, *M* = 3.19, *SD* = 1.20; Achour et al., 2021) measured a generalized tendency to attribute major events to hidden, malevolent forces (e.g., “Politicians and other leaders are only puppets of hidden powers”). *Anti-Migration Attitudes* (4 items; α = 0.78, *M* = 3.10, *SD* = 1.23; Kleinert, 2024) assessed perceived threat from migration to national culture and safety (e.g., “The German culture is threatened by migrants”). *Cavalier Humor Beliefs* (5 items; α = 0.77, *M* = 3.52, *SD* = 1.12; Hodson et al., 2010; Schmid and Greipl, 2025) captured the endorsement of humor tolerance and light-heartedness toward potentially offensive jokes (e.g., “Sometimes people need to relax and realize that a joke is just a joke”; the item “People should try to tell jokes that don't put others down” was excluded to improve reliability). All items were presented in randomized order and scaled from 1 (“strongly disagree”) to 5 (“strongly agree”).

### 6.3 Analysis

**Figure 1:**
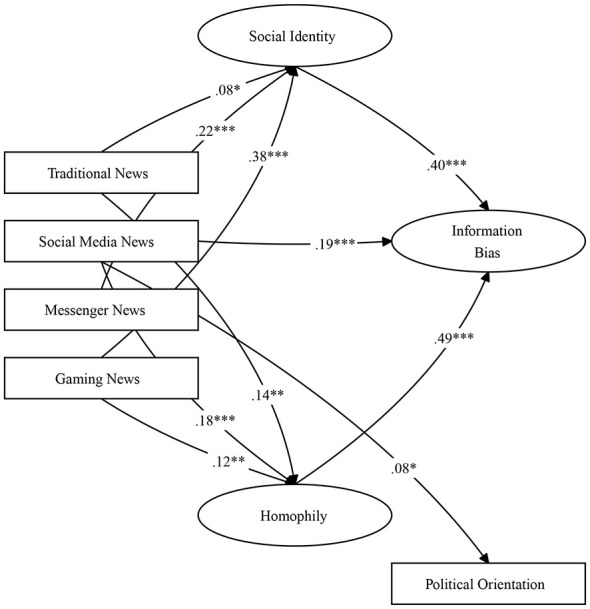
First part (step 1 and step 2) of the sequential structural equation model linking online news use, social–epistemic mediators and political orientation. Standardized path coefficients are displayed. Significant associations are indicated with asterisks (*p* < 0.05, *p* < 0.01, *p* < 0.001). This part shows the associations of news use across different online environments (traditional, social media, messenger, and gaming) with the three factors of the social identity bubble reinforcement scale social identity, homophily, and information bias. News use in low-moderated but highly collective contexts (messenger and gaming) exhibited stronger associations with social identity, while social media news use is associated with information bias directly. Rectangles represent manifest variables, and ellipses represent latent variables.

**Figure 2:**
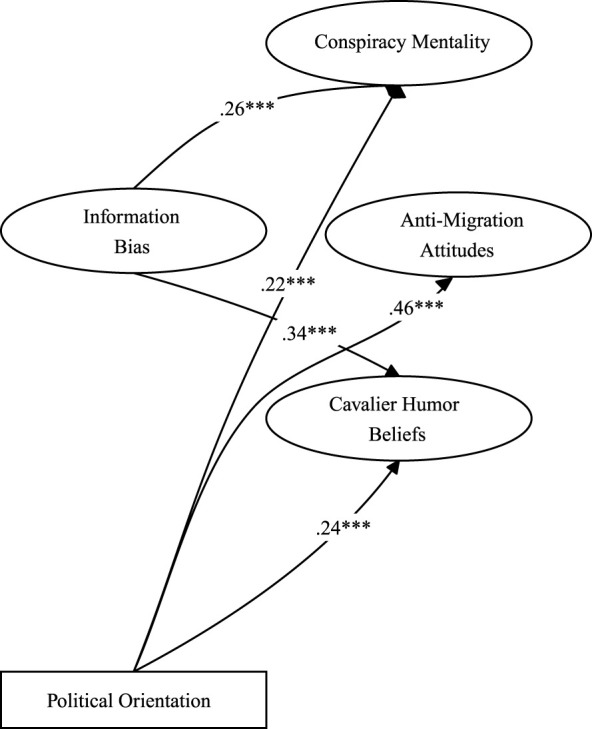
Second part (step 3) of the sequential structural equation model linking social–epistemic mediators and political orientation to radicalization-relevant dispositions. Standardized path coefficients are displayed. Significant associations are indicated with asterisks (*p < 0.05, **p < 0.01, ***p < 0.001). This part shows how Information bias and political orientation are differentially associated with conspiracy mentality, cavalier humor beliefs and anti-migration attitudes. Information bias is related to conspiracy mentality and cavalier beliefs but not anti-migration attitudes. Political orientation was included as a covariate positively associates with all three outcome constructs. Rectangles represent manifest variables, and ellipses represent latent variables.

To test the proposed sequential mediation framework, we estimated a latent structural equation model (SEM) using R (R Core Team., 2025) and the lavaan package (Rosseel, 2012). The model examined whether news use across different online environments—traditional media, social media, messenger apps, and gaming platforms—relate to radicalization-relevant dispositions. The model followed a sequential mediation logic across three stages: (Step 1) news use in specific environments was associated with social identity and homophily; see Figure 1 (Step 2) social identity and homophily were associated with information bias; see Figure 1 and (Step 3) information bias served as the proximal epistemic correlate of belief dispositions, such as conspiracy mentality, anti-migration attitudes, and CHB; see Figure 2. Consistent with the IBRS framework, group-based information bias was modeled as theoretically downstream of identity and homophily (Kaakinen et al., 2020). All four news environments (traditional, social media, messenger, and gaming) entered the model as distinct manifest correlates in all stages. Demographic covariates—age, gender, education, migration background, and political orientation—were included as controls associated with all outcome variables. All indirect effects were computed via bootstrapped confidence intervals (5,000 samples). Sequential indirect associations were calculated for each news environment and outcome combination. Models were estimated using maximum likelihood and full information maximum likelihood (FIML) for missing data. The fit of the hypothesized structural equation model was compared to that of a reverse-specified model to assess the plausibility of the assumed directional relationships. The hypothesized model showed an acceptable to good fit to the data (CFI = 0.904, TLI = 0.885, RMSEA = 0.050, SRMR = 0.054) and substantially lower information criteria (AIC = 48,981.36; BIC = 49,516.82) compared to the reverse model (CFI = 0.906, TLI = 0.891, RMSEA = 0.056, SRMR = 0.065; AIC = 61,187.28; BIC = 61,644.91). Although the reverse model yielded marginally higher incremental fit indices (CFI, TLI), these differences were negligible. In contrast, the hypothesized model demonstrated better fit in terms of RMSEA and SRMR, as well as markedly lower AIC and BIC. Taken together, these results provide robust support for the hypothesized directional specification over the reverse model. To evaluate the structural validity of the measurement model and the German adaptation of IBRS, we conducted a Confirmatory Factor Analysis using again Maximum Likelihood estimation and FIML. The measurement model included six latent constructs: social identity, homophily, information bias, conspiracy mentality, anti-migration attitudes, and cavalier humor beliefs (CHB). Results indicated an acceptable to good fit to the data: χ*2* (194) = 681.47, *p* < 0.001; CFI = 0.928; TLI = 0.914; RMSEA = 0.055 [90% CI (0.051, 0.060)]; and SRMR = 0.055. All standardized factor loadings were significant (*p* < 0.001). Loadings for the IBRS subscales—the primary focus of the adaptation—were substantively high, ranging from 0.79 to 0.85 for social identity, 0.73 to 0.76 for homophily, and 0.49 to 0.64 for information bias. These results confirm that the latent constructs are well-defined and that the German version of the IBRS maintains a factor structure consistent with the original scale.

## 7 Results

For an explication of the full results, see Table 1. The sequential mediation model showed acceptable fit to the data, χ^2^(348) = 912.99, *p* < 0.001, χ*2/df* = 2,36, CFI = 0.93, TLI = 0.91, RMSEA = 0.045 [90% CI (0.041, 0.048)], and SRMR = 0.051.

**Table 1:** Direct associations from the structural equation model.

Dependent	Predictor	β	SE	*z*	95% CI LL	95% CI UL	*p*
Anti-migration attitudes	Information bias	−0.061	0.063	−0.960	−0.184	0.063	0.337
	Traditional news	0.049	0.037	1.332	−0.023	0.122	0.183
	Social Media news	0.063	0.042	1.496	−0.020	0.145	0.135
	Messenger news	0.041	0.042	0.962	−0.042	0.123	0.336
	Gaming news	0.013	0.045	0.284	−0.076	0.101	0.777
	Age	**0.133**	0.036	3.756	0.064	0.203	<0.001^***^
	Gender	0.009	0.036	0.234	−0.063	0.080	0.815
	Education	**−0.161**	0.035	−4.602	−0.229	−0.092	<0.001^***^
	Migration	**−0.089**	0.034	−2.615	−0.155	−0.022	0.009^**^
	Political orientation	**0.459**	0.039	11.870	0.383	0.535	<0.001^***^
Cavalier humor beliefs	Information bias	**0.339**	0.071	4.761	0.199	0.478	<0.001^***^
	Traditional news	0.033	0.042	0.787	−0.049	0.114	0.431
	Social Media news	−0.046	0.050	−0.924	−0.144	0.052	0.356
	Messenger news	−0.004	0.049	−0.071	−0.100	0.093	0.943
	Gaming news	−0.067	0.050	−1.343	−0.164	0.031	0.179
	Age	**0.149**	0.038	3.927	0.075	0.223	<0.001^***^
	Gender	**−0.159**	0.038	−4.154	−0.233	−0.084	<0.001^***^
	Education	−0.033	0.037	−0.899	−0.106	0.039	0.369
	Migration	0.002	0.039	0.059	−0.073	0.078	0.953
	Political orientation	**0.237**	0.037	6.329	0.163	0.310	<0.001^***^
Conspiracy mentality	Information bias	**0.264**	0.066	4.012	0.135	0.392	<0.001^***^
	Traditional news	**−0.107**	0.039	−2.730	−0.184	−0.030	0.006^**^
	Social media news	0.012	0.045	0.264	−0.076	0.100	0.792
	Messenger news	**0.132**	0.045	2.929	0.044	0.221	0.003^**^
	Gaming news	**0.151**	0.046	3.319	0.062	0.240	<0.001^***^
	Age	**0.138**	0.035	3.909	0.069	0.207	<0.001^***^
	Gender	0.024	0.038	0.629	−0.051	0.098	0.529
	Education	**−0.155**	0.036	−4.310	−0.226	−0.085	<0.001^***^
	Migration	**0.097**	0.036	2.723	0.027	0.166	0.006^**^
	Political orientation	**0.216**	0.037	5.783	0.143	0.289	<0.001^***^
Homophily	Traditional news	**0.138**	0.044	3.120	0.051	0.225	0.002^**^
	Social media news	**0.185**	0.046	4.016	0.095	0.275	<0.001^***^
	Messenger news	0.077	0.050	1.549	−0.020	0.174	0.121
	Gaming news	**0.118**	0.043	2.752	0.034	0.202	0.006^**^
	Political orientation	−0.063	0.041	−1.539	−0.143	0.017	0.124
Information bias	Social identity	**0.396**	0.066	6.030	0.267	0.525	<0.001^***^
	Homophily	**0.485**	0.065	7.461	0.358	0.613	<0.001^***^
	Traditional news	0.019	0.039	0.483	−0.058	0.096	0.629
	Social media news	**0.192**	0.047	4.107	0.100	0.283	<0.001^***^
	Messenger news	0.012	0.048	0.239	−0.083	0.107	0.811
	Gaming news	0.063	0.047	1.340	−0.029	0.156	0.180
	Political orientation	0.023	0.037	0.605	−0.050	0.095	0.545
Political orientation	Traditional news	−0.005	0.038	−0.144	−0.079	0.068	0.886
	Social media news	**0.076**	0.039	1.958	0.000	0.153	0.050^*^
	Messenger news	0.073	0.046	1.602	−0.016	0.163	0.109
	Gaming news	0.080	0.047	1.697	−0.012	0.173	0.090
Social identity	Traditional news	**0.078**	0.035	2.246	0.010	0.147	0.025^*^
	Social media news	0.025	0.039	0.641	−0.051	0.101	0.522
	Messenger news	**0.224**	0.044	5.039	0.137	0.311	<0.001^***^
	Gaming news	**0.375**	0.038	9.812	0.300	0.450	<0.001^***^
	Political orientation	−0.016	0.035	−0.460	−0.086	0.053	0.646

Standardized betas are shown. Significant effects are indicated with asterisks and bold values (^*^p < 0.05, ^**^p < 0.01, ^***^p < 0.001). Variance (*R*^2^) explained for endogenous variables: information bias = 0.79; conspiracy mentality = 0.31; anti-migration attitudes = 0.29; CHB = 0.19.

### 7.1 Direct correlates of online news use

Digital news environments exhibited significant direct associations with radicalization-relevant dispositions. Specifically, even when controlling for identity-scaffolded information bias and ideological orientation, news use in high-collectivity environments remained robustly correlated with conspiracy mentality. Messenger news use showed a significant positive direct association on conspiracy mentality (β = 0.13, *p* = 0.003), as did gaming news use (β = 0.15, *p* < 0.001). Conversely, traditional news use was negatively associated with conspiracy mentality (β = −0.11, *p* = 0.006). No significant direct associations were found between platform-specific news use and anti-migration attitudes or cavalier humor beliefs (all *p* > 0.18).

#### 7.1.1 Social Identity, homophily and information bias

News use across platforms showed distinct associations with the identity-based mediators. News use via traditional media (β = 0.08, *p* = 0.025), messenger platforms (β = 0.22, *p* < 0.001), and particularly gaming platforms (β = 0.38, *p* < 0.001) was positively associated with social identity, whereas news use on social media was unrelated (β = 0.03, *p* = 0.52).

For homophily, the tendency to prefer like-minded others, significant positive associations emerged for traditional (β = 0.14, *p* = 0.002), social media (β = 0.19, *p* < 0.001), and gaming (β = 0.12, *p* = 0.007) news use. In contrast, messenger news use was not significantly associated with homophilic orientation (β = 0.08, *p* = 0.12), suggesting that messaging spaces, while t chracterized by intense social interactions, they do not necessarily go hand in hand with a strengthening of perceived preferences for similarity.

Both social identity (β = 0.40, *p* < 0.001) and homophily (β = 0.49, *p* < 0.001) were strongly related to information bias (*R*^2^ = 0.79), the epistemic tendency to privilege identity-congruent information. Additionally, social media news use was directly associated with information bias (β = 0.19, *p* < 0.001), whereas other news environments did not show direct associations (all *p* > 0.10).

Thus, news exposure in particular digital contexts (especially gaming and messenger spaces) was positively associated with social identity and homophily, which in turn was positively related to identity-scaffolded information bias.

#### 7.1.2 Non-normative belief dispositions and political orientation

In the final structural stage, information bias showed robust positive associations with conspiracy mentality (β = 0.26, *p* < 0.001) and CHB (β = 0.34, *p* < 0.001), whereas political orientation showed somewhat smaller associations (β = 0.22, *p* < 0.001 and (β = 0.24, *p* < 0.001, respectively). In contrast, anti-migration attitudes were strongly associated with political orientation (β = 0.46) but not with information bias (β = −0.06, *p* = 0.34). These findings indicate that epistemic filtering processes are more closely linked to conspiratorial and playfully transgressive worldviews rather than explicit xenophobia. Political orientation was associated with higher levels of all three belief dispositions, indicating ideological consistency across outcome domains.

When controlling for mediation, direct associations of platform-specific news use remained for several outcomes. Traditional news use was negatively associated with conspiracy mentality (β = −0.11, p = 0.006), whereas messenger (β = 0.14, *p* = 0.003) and gaming news use (β = 0.15, *p* = 0.002) were positively associated with it. Social media news use was not directly associated with conspiracy mentality (β = 0.01, *p* = 0.78). None of the platform types were significantly linked to anti-migration attitudes or cavalier beliefs once information bias was accounted for (all *p* > 0.18).

#### 7.1.3 Sequential indirect associations

Bootstrapped indirect associations revealed a consistent pattern of sequential mediation linking news exposure through the epistemic chain of social identity and homophily to information bias and finally to belief outcomes; see Table 2. Specifically, significant indirect associations emerged for conspiracy mentality through this sequence for traditional (β = 0.02, *p* = 0.033), social media (β = 0.05, *p* = 0.002), messenger (β = 0.02, *p* = 0.039), and gaming (β = 0.05, *p* = 0.002) news use. An analogous pattern was observed for cavalier humor beliefs, where indirect associations were strongest for social media (β = 0.07, *p* < 0.001) and gaming (β = 0.06, *p* = 0.001), followed by traditional (β = 0.03, *p* = 0.028) and messenger (β = 0.03, *p* = 0.028) contexts. No significant indirect associations of news use on anti-migration attitudes emerged via social identity, homophily, and information bias, suggesting that identity-scaffolded epistemic filtering is largely orthogonal to this form of prejudice in our data.

**Table 2:** Indirect associations (News-use –> identity-scaffolded bias –>radical belief disposition) from the structural equation model.

Dependent	Predictor	β	SE	*z*	95% CI LL	95% CI UL	*p*
Conspiracy mentality	Traditional news	**0.022**	0.010	2.137	0.004	0.045	0.033^*^
	Social media news	**0.054**	0.018	3.059	0.024	0.092	0.002^**^
	Messenger news	**0.020**	0.010	2.062	0.004	0.042	0.039^*^
	Gaming news	**0.045**	0.015	3.108	0.020	0.077	0.002^**^
Anti-migration attitudes	Traditional news	−0.003	0.004	−0.812	−0.013	0.003	0.417
	Social media news	−0.008	0.008	−0.941	−0.025	0.008	0.347
	Messenger news	−0.003	0.003	−0.853	−0.011	0.003	0.393
	Gaming news	−0.006	0.007	−0.898	−0.023	0.006	0.369
Cavalier humor beliefs	Traditional news	**0.027**	0.012	2.200	0.006	0.054	0.028^*^
	Social media news	**0.066**	0.019	3.483	0.032	0.106	<0.001^***^
	Messenger news	**0.025**	0.011	2.197	0.005	0.049	0.028^*^
	Gaming news	**0.055**	0.016	3.475	0.028	0.089	0.001^**^

Standardized betas are shown. Significant effects are indicated with asterisks and bold values (^*^p < 0.05, ^**^p < 0.01, ^***^p < 0.001).

#### 7.1.4 Control variables

Among controls, age was positively associated with anti-migration attitudes (β = 0.13, *p* < 0.001), conspiracy mentality (β = 0.14, *p* < 0.001) and CHB (β = 0.15, *p* < 0.001), while education showed similar negative associations with these outcomes except for CHB. Migration background was negatively associated with anti-migration attitudes (β = −0.09, *p* = 0.009) and positively with conspiracy mentality (β = 0.10, *p* = 0.006).

## 8 Discussion

The present study examined how the consumption of political news in distinct online environments relates to radicalization-relevant dispositions and whether these relationships are mediated by socially scaffolded epistemic style. Integrating concepts from social identity theory, epistemic psychology, and digital communication research, we proposed that less moderated, more interactive and community-oriented platforms, such as gaming spaces and messenger apps, provide environments that may foster group-based identification and homophily, which jointly drive selective information processing termed identity-scaffolded information bias. This epistemic tendency, we hypothesized, would serve as a cognitive-affective bridge between digital news use and the formation of conspiratorial and norm-violating belief orientations.

Our results provide empirical evidence that may speak in favor of this sequential pattern: news use on messenger and gaming platforms was positively associated with social identity, while social media news use was directly associated with information bias. Both social identity and homophily were robustly associated with information bias, which in turn was associated with conspiracy mentality and cavalier humor beliefs (CHB). Indirect associations from platform-specific news use to belief outcomes were strongest for gaming and social media contexts. Anti-migration attitudes, in contrast, did not show a sequential indirect association over identity-scaffolded epistemic style, but was directly associated with political orientation. These results underscore the idea that the epistemic impact of online news is not only linked to message content but also to the socio-technical architectures and context where individuals engage with political information.

### 8.1 The social-epistemic scaffolding of belief

The finding that social identity and homophily were significantly associated with information bias, explaining the majority of its variance, supports the view that epistemic filtering is socially scaffolded rather than individually generated. Rather than a generalized instantiation of identity-protective cognition (Kahan, 2017), our results suggest a relational, identity-scaffolded and environment-dependent mechanism. The IBRS constructs were differentially associated with platform-clusters, which supports the notion of an environmental prosthesis, where epistemic styles are structurally co-created by digital architectures rather than applied universally. Individuals may not merely fail to reason but outsource validation to the group, a mechanism that in our data related to group-dependent beliefs such as conspiracy mentality and CHB, but not to anti-migration attitudes, whereby the latter can still be more broadly considered identity-relevant. Furthermore, our results align with coherence-based reasoning accounts (Simon and Read, 2025). This pattern is remarkably stable because individuals do not evaluate evidence in isolation; they adjust interpretations and weights to achieve maximal global coherence among their beliefs and social commitments. In high-collectivity online environments, social identity and homophily provide the affective constraints, while group-information bias provides the epistemic filter. Together, they yield a coherent, self-reinforcing worldview that sustains conspiracy mentality while leaving more perception-based or ideologically anchored anti-migration attitudes unaffected.

### 8.2 Environment-specific patterns

An important finding concerns the differential socio-epistemic profiles of digital environments. Gaming platforms showed the strongest overall associations, uniquely combining social identity and homophily with radical belief dispositions. Because gaming communities often blur entertainment with identity performance (Newhouse and Kowert, 2025), political information encountered there may be interpreted within a context of affective play, creating a fertile ground for conspiratorial thinking and disparaging humor.

Messenger platforms were similarly linked to identity-related processes but were less strongly tied to homophily. Since news exchange in messaging apps typically occurs within strong-tie networks where trust is high, but ideological diversity may persist, identity affirmation—appears to be the primary correlate of epistemic bias.

In contrast, the use of social media platforms (e.g., TikTok, Instagram) was directly associated with information bias without necessarily strengthening identity or homophily. This likely reflects how some platforms like TikTok do not primarily rely on stronger social ties or are less oriented toward group-based discussions than messenger and (gaming) forums. Alternatively, this suggests that algorithmic curation may act as a structural substitute for social scaffolding, facilitating epistemic segregation at the level of attention and feed composition even in the absence of explicit social bonds.

Traditional media consumption, conversely, showed a negative direct association with conspiracy mentality. This underscores the stabilizing role of editorial gatekeeping and journalistic standardization against identity-driven mechanisms. However, the presence of indirect associations suggests that if an online identity-scaffolded bias is already established, even reliable news might be reinterpreted to reinforce radical ideas.

In sum, platforms that encourage social connectedness, identity affirmation, and shared meaning-making appear particularly relevant to belief dispositions that challenge socio-epistemic norms. This is in line with predictions from affordance literature on online radicalization (Schulze et al., 2024). It is also consistent with the broader body of theoretical and comparative research observing identity enactment, maintenance and reinforcement in online spaces, especially from extremist actors (Phadke and Mitra, 2024; Törnberg and Törnberg, 2022; Vaux et al., 2021), as well as the perspective of an identity turn in political communication (Lane et al., 2026).

### 8.3 From information bias to epistemic dispositions

Identity-scaffolded bias was positively associated with conspiracy mentality and CHB, confirming that such beliefs may require ongoing social validation and are sustained within “identity bubbles”. Conspiracy thinking exemplifies a state of epistemic dependence (e.g., the reliance on the knowledge and testimony of others; Nguyen, 2020), while CHB reflects a group-reinforced belief that dampens the harmful component of disparaging humor (Hodson et al., 2010) and points to how playfulness of online discourse can become a subtle vector for ideological normalization (Schmid, 2025). These dispositions thrive on validation rather than veracity, functioning as socially sustained epistemic styles that erode trust in shared facts.

Anti-migration attitudes, in contrast, seemed rather independent of socio-affective or epistemic scaffolding. Thus, it appears to be an ideological content dimension anchored in salient social categories observable in everyday life. These attitudes may often rely on perceptual, even threat-based immediacy of visible differences or cultural or economic competition (Hjorth, 2020; Meuleman, 2018). While anti-migration attitudes are not a universal right-wing attribute across political systems (Leykin and Gorodzeisky, 2024), it is a stable component of conservative/right-wing ideological constellations, especially where right-wing authoritarianism, social dominance orientation and nationalism are salient (Pellegrini, 2023). These aspects suggest that anti-migration attitudes may represent already “stabilized knowledge” or may additionally provide a form of self-sustaining validation that does not require the same level of socio-epistemic scaffolding like conspiracy mentality or CHB.

This empirical boundary highlights a central conceptual distinction between ideology as content and epistemic style as a mode of validation. This distinction is supported by the structure of our model: for conspiracy mentality, identity-scaffolded information bias emerged as a significant correlate alongside political orientation. While both factors contribute to the variance, the high explained variance in information bias suggests that this epistemic filter is itself a product of the social scaffold. In contrast, for anti-migration attitudes, this socio-epistemic mechanism provided no additional explanatory value, leaving political orientation as the primary correlate.

Platform-specific associations highlight that radicalization processes may not uniformly distributed across the digital ecosystem. The same political message may have divergent psychological effects depending on whether it circulates in a personalized social feed, a private messenger group, or a gaming forum. The structure of social interaction, alongside the ideology of content, thus appears central to epistemic outcomes. However, while our model identified a significant indirect association through identity-scaffolded bias, the robust direct association of gaming and messenger news use on conspiracy mentality suggests that these socio-epistemic mechanisms do not solely account for the observed associations. Other factors, including a combination of gaming- culture-related elements and individual-level factors may underlie the appeal of radical and extremist narratives as attractors for disenfranchised gamers (Wells et al., 2024). Future research is required to further disentangle these mechanisms.

### 8.4 Identity-scaffolded epistemology in the digital age

The findings underscore the necessity of viewing online radicalization not merely as a process of exposure to extremist content but as the social construction of epistemic worlds. Digital environments, particularly those that combine interaction, entertainment, and privacy, can function as spaces where users continuously test and reaffirm their sense of belonging through interpretive acts.

In this sense, (dis)trust in shared facts emerges not only from ideology but also from the social infrastructure of meaning-making. Gaming, messaging, and social media platforms provide emotional and cognitive affordances, such as validation, immediacy, and play, where political information is more readily integrated with identity work. Interventions focused solely on debunking may fail because they neglect the social and affective foundations of these socio-epistemic processes. Effective countermeasures must foster cross-group contact, reflexivity, and offline opportunities for identity affirmation.

### 8.5 Limitations

Several limitations warrant consideration. First, the data are cross-sectional, precluding causal inference. While our sequential mediation model is consistent with theoretical expectations and demonstrated statistically superior fit compared to a reverse-path specification, the possibility of reverse causality, whereby individuals with pre-existing conspiratorial or CHB tendencies actively seek out congruent environments, cannot be ruled out. Aside from directionality, digital radicalization may also be characterized by a mutual dependency. In this view, identity-scaffolded epistemic styles and platform-specific news use may reinforce each other over time, gradually stabilizing radical belief dispositions through iterative socio-technological feedback. This is important for a clearer distinction between strategic and organic identity dynamics in these spaces in future works. Longitudinal or experimental designs would be required to validate the temporal ordering of effects. Second, the study relied on self-reported measures of platform and news use, which may be subject to recall bias or social desirability effects. Future research could complement this with digital trace data. Third, while the model included a broad range of control variables, it did not explicitly account for other personality traits (e.g., openness, need for closure) or motivational variables (e.g., uncertainty tolerance) that may moderate identity-based epistemic processes. Fourth, these analyses are based on relatively broad platform categories, without differentiating between specific services such as individual gaming forums (e.g., Steam vs. Discord) or messenger applications (e.g., Telegram vs. WhatsApp). Such platforms likely exhibit important internal heterogeneity, including differences in moderation practices, degrees of algorithmic curation, and forms of user interactivity. Accordingly, while the present findings capture higher-level structural patterns across categories, more fine-grained platform-level analyses may reveal additional nuances in how these mechanisms unfold. Fifth, we distinguish between ideology and identity-scaffolded bias, but our approximation through political orientation is limited and should be operationalized more comprehensively in the future. Specifically, key ideological drivers such as Social Dominance Orientation and Right-Wing Authoritarianism could help to isolate the unique variance of epistemic style from stable personality traits. Sixth, we explicitly acknowledge the psychometric constraints of the IBRS Information Bias subscale (α = 0.59). While our latent SEM approach mathematically corrects for manifest-level measurement error by deattenuating the structural paths, this specific subscale must be interpreted cautiously. It functions within our model as an exploratory proxy for identity-congruent credibility judgments rather than a granular psychometric measure of cognitive bias. Future research aiming to isolate the exact cognitive mechanics of epistemic scaffolding should utilize more extensive and robust behavioral measures of group-based information selectivity. Finally, the study was conducted in the specific sociocultural context of Germany, characterized by relatively strict content moderation practices, which may limit generalizability. The sample was further restricted to young adults—a highly relevant group in the study context, likely to participate in the examined platform environments daily. However, this restriction limits external validity across age cohorts. It remains an open question to what extent the proposed pattern generalize to older populations, who not only differ in their platform use but may also be more strongly embedded in stable social groups and established ideological orientations, potentially reducing the relative importance of platform-based identity scaffolding. More broadly, future research should examine whether these patterns hold across different political cultures and regulatory environments, where platform governance and norms of political communication may vary substantially.

## 9 Conclusion

News encountered in online environments allowing or even supporting identity enactment, particularly gaming and messenger platforms, appears to be positively associated with the social and affective foundations of belief formation, including identity alignment, homophily, and ultimately information bias. These pattern, in turn, are related to conspiratorial and norm-relativizing worldviews that challenge collective epistemic integrity and can support radicalization dynamics.

The study advances an integrated model in which identity-scaffolded information bias, a group-based trust in identity-congruent information, links distinct online news environments to conspiratorial and norm-relativizing epistemic styles. Importantly, it highlights a differentiation between ideological content and epistemic style. Our integrated model suggests that adopting non-normative beliefs is not merely the consequence of consuming extremist content, but a gradual, socially scaffolded facilitation of epistemic practice. The manifestation of bias is a result of psychological need for group affiliation, neurobiological reward systems, and the amplification mechanisms of modern media and technology. This contributes to our understanding of how social identity and epistemic selectivity jointly mediate the link between digital news exposure and radical belief orientations. By identifying online pathways to non-normative beliefs associated with radicalization dynamics, this study provides actionable insights for digital interventions that move beyond content-moderation toward addressing the socio-epistemic affordances of online spaces. Safeguarding democratic discourse in the digital age requires attention to the social contexts of belief formation. Understanding how online environments cultivate epistemic practices through collectivity may provide a window into the everyday mechanisms by which individuals' belief dispositions are stabilized as they come to know, doubt, and belong.

## Data Availability

The datasets presented in this study can be found in online repositories. The names of the repository/repositories and accession number(s) can be found in the article/supplementary material.

## Footnotes

1 Operationalizing “identity” in digital contexts is complex, as individuals hold multiple, overlapping, and sometimes conflicting identities (Roccas and Brewer, 2002), shaped by broader histories and power structures (Hornsey, 2008). We approximate identity through the latent dimensions adapted from Kaakinen et al. (2020) which captures the unspecific salience of an online social identity.

2 Although conspiracy mentality and CHB are important correlates of radicalization in other work, our sample consisted of general-population young adults, and we did not measure extremist behavior or support for violence. We therefore interpret our findings as speaking to radicalization-relevant epistemic styles rather than to extremism *per se*.
